# Deciphering the Relationship Between the Trough Concentration of Posaconazole and Its Efficacy and Safety in Chinese Patients With Hematological Disorders

**DOI:** 10.3389/fphar.2020.575463

**Published:** 2020-10-08

**Authors:** Meng-Meng Jia, Qi-Wen Zhang, Zi-Fei Qin, Run-Qing Lu, Xue-Ke Tian, Jing Yang, Xiao-Jian Zhang

**Affiliations:** ^1^Department of Pharmacy, The First Affiliated Hospital of Zhengzhou University, Zhengzhou, China; ^2^Henan Key Laboratory of Precision Clinical Pharmacy, Zhengzhou University, Zhengzhou, China; ^3^Department of Hematology, The First Affiliated Hospital of Zhengzhou University, Zhengzhou, China

**Keywords:** posaconazole, invasive fungal infection, pharmacokinetics, drug monitoring, hematological disorders

## Abstract

Posaconazole (PCZ) is effective in preventing and salvage treatment invasive fungal infections in patients with hematologic disorders. However, PCZ displays highly variable individual pharmacokinetics affecting its efficacy and safety. To investigate the correlation between PCZ concentration and efficacy and safety, the following key influencing factors were explored. A total of 285 trough plasma concentrations (C_min_) of 81 Chinese patients receiving PCZ oral suspension for prophylaxis or treatment of invasive fungal infections were collected in this study. The relationships between C_min_ values and clinical response and hepatotoxicity were investigated as well as the incidence of clinical response under different C_min_ values of PCZ with a logistic regression model. The concentration of PCZ showed remarkable differences among patients with haematologic disorders. PCZ C_min_ values of 0.76 and 1.0 µg/mL were both associated with an over 80% probability of successful response to prophylaxis and treatment of fungal infections, respectively. No association between C_min_ values and hepatotoxicity was noted (*P* > 0.05). Gender, albumin, and co-administration of proton pump inhibitor (PPI) were identified as independent factors influencing PCZ C_min_ by multiple linear regression analysis. Furthermore, patients’ C-reactive protein (CRP), albumin, and co-administration of PPI exhibited significant effects on the therapeutic window of patients receiving PCZ for prophylaxis. The plasma concentration is closely associated with therapeutic efficacy of PCZ. It is necessary to adjust the dosing regimens based on PCZ C_min_ to obtain an optimal therapeutic response.

## Introduction

Hematological disorders patients are susceptible to fungal infections. Posaconazole (PCZ) is a new-generation triazole with broad-spectrum antifungal activity against a variety of fungal pathogens. Because it is generally well tolerated and the majority of its adverse side effects are similar to those of other triazoles (Huang et al., 2012; Shen et al., 2013), PCZ is potentially an important agent in the treatment and prevention of invasive fungal infections (IFIs) in hematological disorders patients (Cornely et al., 2007; Ullmann et al., 2007).

PCZ shows significantly variable bioavailability due to erratic absorption, especially with the oral suspension formulation, which is influenced by food, some medications and gastric absorption disorders. Up to 4-fold increase in the exposure of the oral PCZ suspension was reported when administered with food (Krishna et al., 2009). Conversely, medications that increase gastric pH, such as proton pump inhibitors (PPIs) and histamine H_2_-blockers, have been shown to decrease PCZ exposure to a considerable extent (Dolton et al., 2012). However, conclusive clinical studies investigating the effects of gender and inflammation on PCZ metabolism are lacking, although a few differences have been reported in terms of clinical outcome and physiological and pharmacological effects between men and women undergoing PCZ (Allegra et al., 2017; Jeong et al., 2018). Besides, clinically significant effect of C-reactive protein (CRP) on PCZ concentration was not observed in a prospective study by Märtson et al. (2019a).

Moreover, a significant correlation between clinical response and PCZ concentrations has been demonstrated, and findings from these studies have shown that monitoring PCZ concentration is necessary to improve antifungal effect (Dolton et al., 2012; Moore et al., 2015). However, real-world data in this field is limited, especially in Asian populations. One Chinese study suggested that the concentration of PCZ affects the probability of response to prophylaxis of fungal infections, but factors, other than PPIs, affecting PCZ concentrations were not investigated so far (Li et al., 2020). One South Korea study suggested that hypoalbuminemia influences PCZ concentrations, but the concentration-to- response relationship was not explored (Oh et al., 2020). The aim of present study was to examine the impact of PCZ concentration on the clinical response and hepatotoxicity in Chinese patients with hematological disorders, and to investigate potential factors that may affect PCZ concentration.

## Methods

### Patient Enrollment and Data Collection

This was a retrospective observational study performed in The First Affiliated Hospital of Zhengzhou University (Zhengzhou, China). Adult patients (age, ≥18) with hematological disorders taking PCZ suspension for treatment or prophylaxis of fungal infections and performing at least one PCZ concentration measurement during therapy between June 2018 and December 2019 were enrolled in this study. Data was gathered from patients’ medical records and laboratory reports, which included patients’ demographics, clinical data on outcomes of therapy, adverse events, and details of PCZ therapy. In addition, other laboratory test data including CRP, albumin, alkaline phosphatase (ALP), aspartate aminotransferase (AST), alanine aminotransferase (ALT), γ-GT (gamma-glutamyl transferase), and bilirubin values were also collected. The biological data evaluated in **Table 3** was the one collected on the day blood plasma concentration was measured, and the clinical data, including potential drug interactions, was collected during the 5 days before blood plasma concentration was measured.

### Measurement of PCZ Plasma Concentration

The blood samples for measuring PCZ C_min_ were collected for routine care. Only patients having achieved steady-state were included in the analysis, and PCZ was considered to have achieved a steady-state plasma concentration after at least 7 days of dosing. PCZ concentrations were measured by a validated acquity ultra high-performance liquid chromatograph-tandem mass spectrometry method (UPLC- MS/MS, Waters, United States). The analyte and internal standard, PCZ d4, were extracted from human plasma *via* protein precipitation pretreatment and separated on a Waters XBridge BEH C18 (2.1 mm × 50 mm, 1.7 µm) with a temperature set at 40°C. The mobile phase consisted of 0.1% formic acid water (solvent A), and 0.1% methanol formate (solvent B). The gradient elution program was linear from 5 to 90% B for 0–3.0 min; linear from 90 to 5% B for 3.0–3.1 min and then held at 5% B for 0.9 min. The flow rate was 0.4 mL/min, and the injection volume was 3 µL. The electrospray ion source (ESI) was employed as the ion source. The MS parameters were set as follows: a 110°C source temperature, a 650 L/h desolvation gas flow rate, a 350°C desolvation temperature, a 50 L/h cone gas flow rate, and a 3200 V capillary voltage. MS acquisition was performed in multiple reaction monitoring mode and the mass transitions were 701.2→127 for PCZ, and 705.2→127 for PCZ d4. This method was developed and validated according to FDA guidelines. The intra- and inter-assay coefficients of variation of 3.63–4.02%, 2.37–3.65%, and 1.19–3.02% for low, medium, and high levels of internal quality controls, respectively).

### Diagnostic Criteria

Only patients with a PCZ treatment regimen >14 days were used to assess clinical response. Efficacy was evaluated at the end of PCZ treatment in patients with IFIs by clinical and microbiological responses. IFIs were classified in accordance with previously defined criteria (Lerolle et al., 2014). For patients who received PCZ for the treatment of a fungal infection, a successful outcome was defined as partial improvement or improvement of clinically significant signs and symptoms, improvement or resolution of radiological signs of infection and evidence of microbiological cure; otherwise, patients were classified as clinical failure. Patients receiving PCZ prophylaxis without breakthrough infection were considered to have successful therapy. Hepatotoxicity was defined as an increase in ALT or total bilirubin of 3 or 1.5 times the upper limit of normal, respectively (Gomes et al., 2014).

### Data and Statistical Analysis

Descriptive statistics included the mean, standard deviation, and median. The categorical variables were expressed as frequency and percentage. Baseline demographics and plasma PCZ concentrations differences between groups were analyzed by using the chisquare test, ManneWhitney U test, or KruskaleWallis test, as appropriate. The association of C_min_ with efficacy and safety was analyzed by logistic regression to obtain the target PCZ trough plasma concentration range. The logistic regression model was calculated according to equation (1), the P_i_ value reflected the probability of PCZ related response in the individual and was calculated as equation (2). *θ*_1_ is the baseline probability of response; *θ*_2_ is the logarithm of the contribution ratio of drug exposure; *λ*_i_ is the logit value, the natural logarithm of odds ratio.

Considering the large variations in PCZ daily doses, we further normalized the PCZ concentrations to dose and investigated their correlation with various potential risk factors. Both univariate and multivariate linear regression analyses were used to identify the relationship between dose normalized PCZ concentration and potential factors. Data analysis was performed with SPSS Statistics for Mac Ver. 26 (SPSS Inc., Chicago, IL). GraphPad Software (GraphPad Prism 7.0) was used for mapping. Values of *P* < 0.05 were considered statistically significant.

(1) $$\lambda_{i}=\theta_{1}+\theta_{2}\times \text{exposure}$$

(2) $$p_{i}=\frac{1}{1+e^{-\lambda_{i}}}$$

## Results

### Patient Characteristics

**Figure 1:**
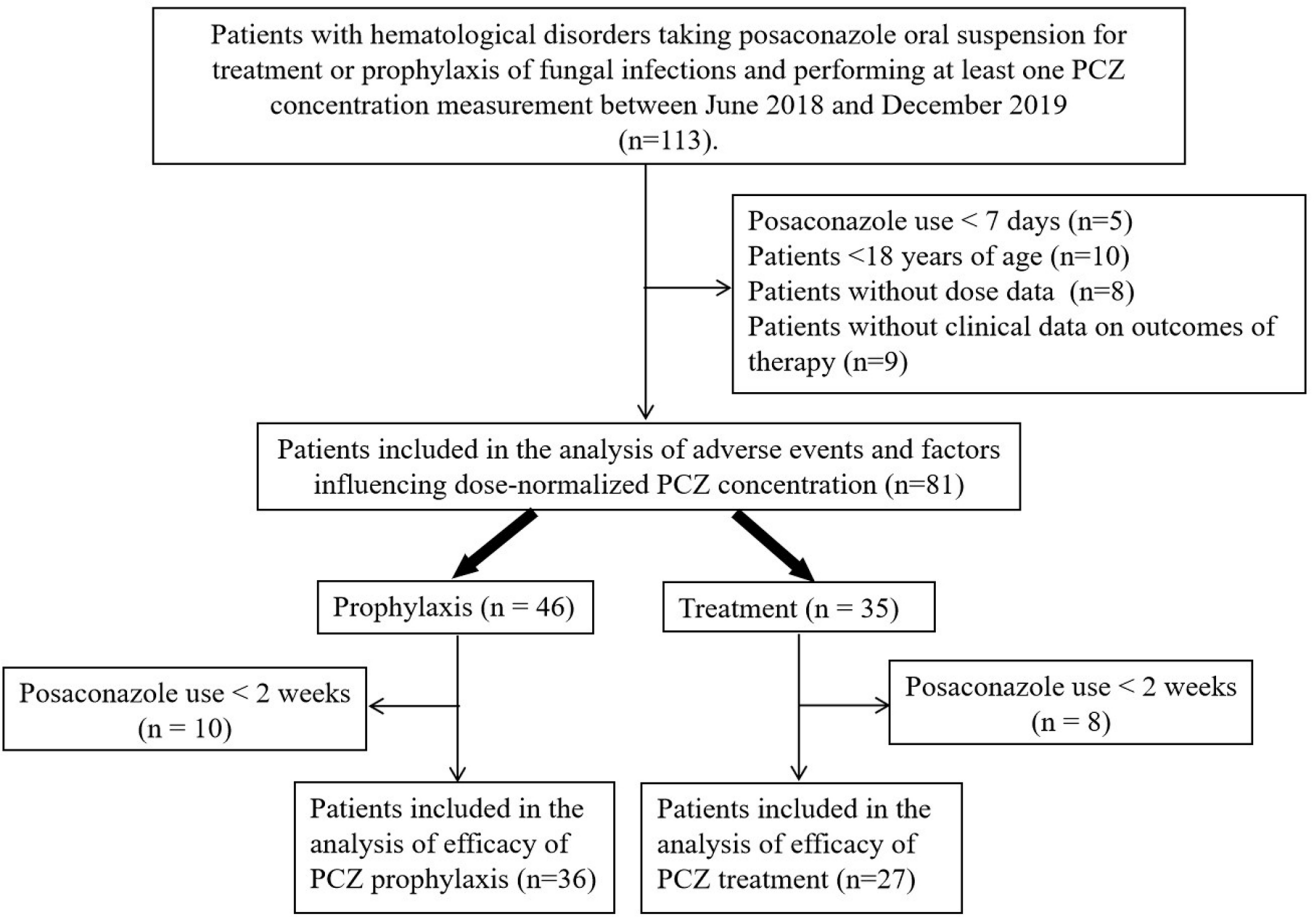
Flow chart of the study.

**Figure 2:**
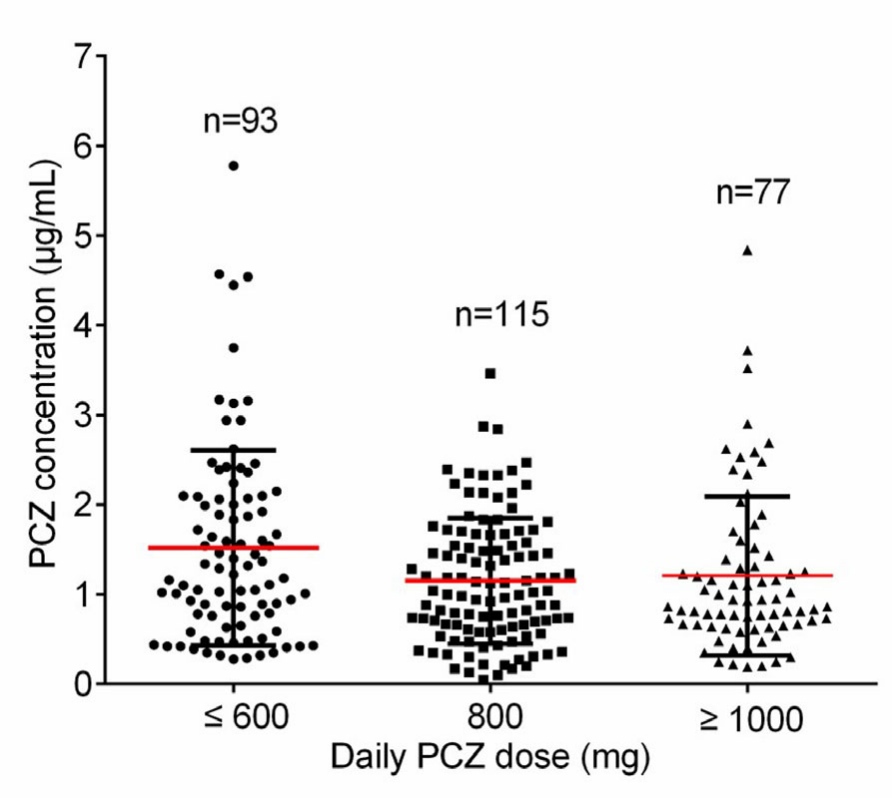
The distribution for PCZ trough concentrations. The horizontal red lines represent the mean value and the lower and upper black lines represent the SD value, respectively.

A total of 113 patients were screened, and only 81 (52 male, 64.2%) were included in the study. The other 32 patients were excluded because of missing medical records or laboratory reports (**Figure 1**). A total of 285 blood samples for PCZ C_min_ were obtained. 46 patients (56.8%) took PCZ for prophylaxis and 35 patients (43.2%) took PCZ for treatment. The demographic and clinical characteristics of the 81 patients are summarized in **Table 1**. 49% (42/81) of the patients received 800 mg/day of PCZ on the first day of treatment. All PCZ concentrations are summarized in **Figure 2**.

**Table 1:** Patient characteristics (n = 81).

Characteristics	Total (n = 81)	Prophylaxis (n = 46)	Treatment (n = 35)
Male	52 (64.2)	26 (56.5)	26 (74.3)
Age (years)	39 (18–81)	34 (16–79)	49 (17–81)
BMI (kg/m^2^)	21.13 (16.05–28.73)	22.03 (16.05–28.73)	22.99 (16.53–27.72)
C_min_, µg/mL	1.03 (0.10–5.78)	1.11 (0.10–5.78)	0.94 (0.15–2.87)
C_min_/dose, µg·ml^-1^·g^-1^	1.30 (0.13–9.63)	1.48 (0.13–9.63)	0.98 (0.13–3.59)
Underlying conditions			
Acute myeloid leukaemia	22 (27.2)	14 (28.6)	8 (22.9)
Myelodysplastic syndrome	9 (11.1)	5 (10.9)	4 (11.4)
Aplastic anemia	5(6.2)	4 (8.7)	1 (2.9)
Acute lymphoblastic leukemia	19 (23.5)	11 (23.9)	8 (22.9)
Non-Hodgkin’s lymphoma	8(9.9)	6 (13.0)	2 (5.7)
Hemophagocytic syndrome	6 (7.4)	4 (8.7)	2 (5.7)
Other	13 (16.0)	8 (17.4)	5 (14.3)

Data is presented as n (%), or as median (range). BMI, Body mass index. C_min_, rough plasma concentration.

### PCZ Concentration and Clinical Outcomes in Prophylaxis

**Figure 3:**
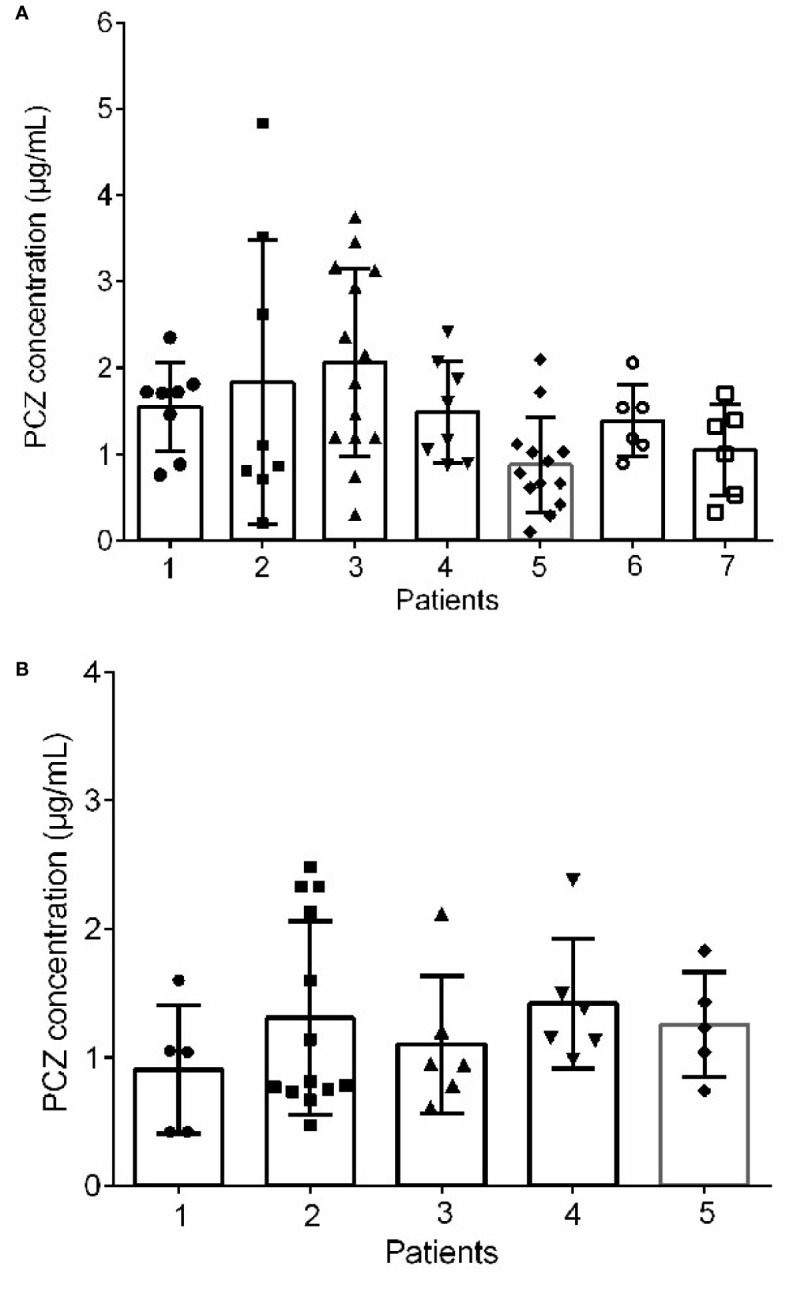
Intra‐and inter-patient variability for patients received 800 mg/day of PCZ suspension in prophylaxis **(A)** and treatment **(B)** groups. The graphs show mean values and SD values.

Forty-six patients (26 male, 56.5%) received PCZ for prophylaxis (190 PCZ samples). In 47.8% (22/46) patients, 600 mg daily was prescribed as a starting dose, and 43.6% (20/46) patients received a starting dose of 800 mg daily. A mean of 4.2 (sd 3.7) blood samples was taken per patient. Large intra- and inter- patient variability for patients receiving 800 mg/day of PCZ suspension who had five or more sample measurements was observed (**Figure 3A**).

**Figure 4:**
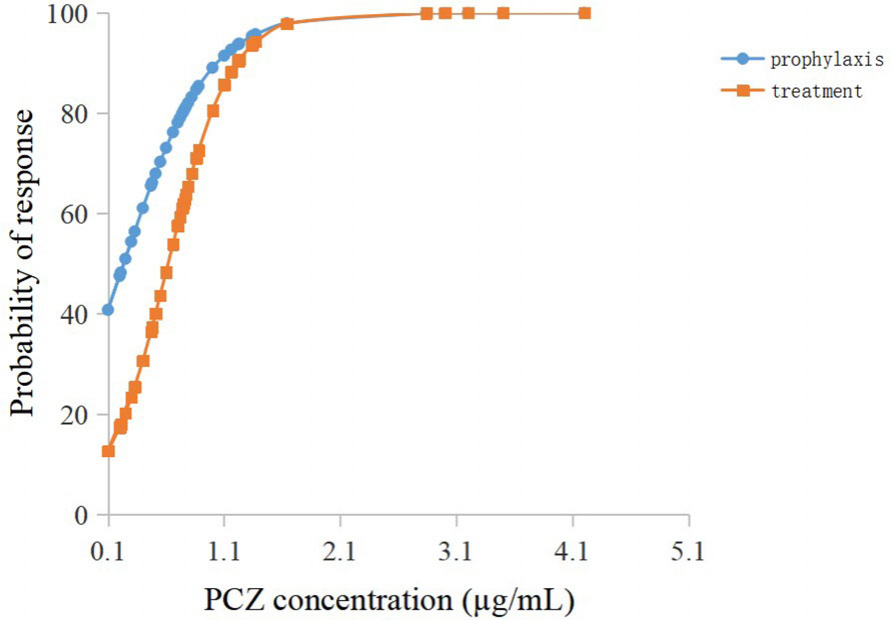
Logistic regression model showing posaconazole trough concentration predicting the probability of successful clinical response of prophylaxis and treatment.

Among the forty-six patients receiving PCZ for the prophylaxis of IFIs, thirty-six eligible patients were identified in the analysis of efficacy of PCZ prophylaxis, ten were excluded because of administration of PCZ less than two weeks. Six of the thirty-six (16.7%) patients developed proven or probable breakthrough IFIs. Logistic regression analysis showed that the PCZ C_min_ correlated well with clinical responsiveness, and PCZ C_min_ values of 0.76 µg/mL was associated with a >80% probability of successful response (**Figure 4**). The quartile analysis of PCZ C_min_ and clinical response indicated that higher plasma concentrations of PCZ were associated with lower clinical failure rates (**Table 2**).

**Table 2:** Association between the PCZ steady-state average plasma concentrations quartiles (Q) and clinical response in the whole population.

Quartile	Prophylaxis (n = 36)			Treatment (n = 27)		
	PCZ concentration(µg/mL)	Clinical success (%)	Clinical failure (%)	PCZ concentration (µg/mL)	Clinical success (%)	Clinical failure (%)
Q1	0.33–0.73	6 (14.3)	3 (7.1)	0.22–0.74	3 (11.1)	3 (11.1)
Q2	0.74–1.10	7 (16.7)	2 (4.8)	0.75–1.05	6 (22.2)	1 (3.7)
Q3	1.11–1.89	8 (19.0)	1 (2.4)	1.06–1.54	5 (18.5)	2 (7.4)
Q4	1.90–4.57	9 (21.4)	0 (0)	1.55–2.87	7 (25.9)	0 (0)

The mortality rate in the prophylaxis group was 8.7% (four patients), three patients had adequate PCZ concentrations (≥0.76 µg/mL), and one patient had one concentration below 0.76 µg/mL. Mortality was not attributed to a fungal infection. It is of note that a single patient had persistently low PCZ concentrations despite several dose modifications, with no potential drug interaction being identified to account for these persistently low levels.

### PCZ Concentration and Clinical Outcomes in Treatment

Thirty-five patients (26 male, 74.3%) received PCZ for treatment (95 PCZ samples). Most patients [22/35 (62.9%)] received a starting dose of 800 mg daily. A mean of 2.7 ± 2.4 blood samples was taken per patient. The interpatient variance was 0.48 and standard deviation 0.70. **Figure 3B** shows intra- and inter-patient variability for patients receiving 800 mg/day of PCZ suspension who had 5 or more samples measured.

Twenty-seven eligible patients were included in the analysis of efficacy of PCZ treatment, eight were excluded because of administration of PCZ less than two weeks. Among the 27 patients receiving PCZ for the treatment of a proven/probable IFI, overall successful treatment response was 77.8% (21/27). Logistic regression analysis showed that PCZ C_min_ values of 1.0 µg/mL was associated with a >80% probability of successful treatment response (**Figure 4**). The incidence of treatment failure in patients who attained C_min_ <1.0 µg/mL was 36.4% (4 IFIs in 11 patients), whereas it was only 12.5% (2 IFIs in 16 patients) in patients who attained C_min_ ≥ 1.0 µg/mL.

### PCZ C_min_ and Adverse Events

During PCZ therapy, 24.7% of patients (20/81) experienced hepatotoxicity. The PCZ plasma concentrations tended to be higher in patients with hepatotoxicity (1.83 ± 1.47 µg/mL) as compared with the patients without hepatotoxicity (1.2 ± 0.83 µg/mL), although these differences were not statistically significant (*P* = 0.07). The mean dosage for patients who experienced hepatotoxicity was not significantly different from that of patients who did not (839.52 ± 236.1 mg vs. 809.52 ± 288.1 mg, *P* = 0.578).

### Factors Influencing Dose-Normalized PCZ Concentration

**Table 3** shows the factors influencing dose normalized plasma PCZ C_min_. The dose normalized PCZ concentration was significantly correlated with patients’ CRP (*P* = 0.002), albumin (*P* < 0.001), alanine transaminase (*P* = 0.017) and PPIs (*P* = 0.001). The patients receiving PPIs had a 25.9% lower mean blood plasma concentration (1.10 µg/mL vs. 1.48 µg/mL, *P* = 0.001). Of the five PPIs, pantoprazole (n = 38) showed to have the most significant effect on PCZ concentration (*P* < 0.001). Lansoprazole (n = 16) had also a significant effect (*P* = 0.004), whereas omeprazole (n = 83), rabeprazole (n = 20) and esomeprazole (n = 3) was not significantly found to influence PCZ concentration (*P* = 0.058, *P* = 0.194 and *P* = 0.179, respectively). Stepwise regression analysis was used to identify independent factors influencing PCZ concentration. As shown in **Table 4**, the gender, albumin, and the co-administration of PPIs were independent influencing factors of PCZ C_min_, contrary to ALT and CRP (*P* = 0.052 and *P* = 079, respectively).

**Table 3:** Univariate analysis of factors influencing dose normalized plasma PCZ concentration.

Parameters	Estimate	95% CI	*P* values
Gender	-0.390	-0.828, -0.047	0.079
Age (years)	-0.004	-0.013, 0.004	0.309
BMI (kg/m^2^)	0.002	0.000, 0.000	0.278
Nausea/emesis	-0.302	-0.704, 0.099	0.139
Diarrhoea	0.101	-0.847, 1.049	0.834
Mucositis	-0.288	-0.936, 0.361	0.383
C-reactive protein(CRP, mg/L)	-0.006	-0.010, -0.002	**0.002**
Creatinine clearance (GFR, mL/min)	0.003	-0.002, 0.007	0.253
Albumin (g/L)	0.032	0.016, 0.048	**<0.001**
Alanine transaminase (ALT, U/L)	0.002	-0.005, 0.224	**0.017**
Aspartate transaminase (AST, U/L)	0.003	-0.007, 0.152	0.233
γ-Glutamyltranspeptidase (γ-GT, U/L)	0.000	-0.002, 0.001	0.598
Alkaline phosphatase (ALP, U/L)	0.000	-0.003, 0.002	0.779
Total bilirubin (TBIL, mmol/L)	0.000	-0.007, 0.006	0.895
Proton pump inhibitor	-0.596	-0.944, -0.248	**0.001**
Omeprazole (n = 83)	0.445	-0.015,0.906	0.058
Rabeprazole (n = 20)	0.570	-0.294, 1.434	0.194
Esomeprazole (n = 3)	1.419	-0.662, 3.500	0.179
Lansoprazole (n = 16)	0.768	0.257, 1.279	**0.004**
Pantoprazole (n = 38)	0.932	0.544, 1.320	**<0.001**
Metoclopramide (n = 30)	-0.342	-0.884, 0.198	0.213

The bold values indicate significant differences (p < 0.05).

**Table 4:** The multiple linear regression analysis for dose- normalized PCZ concentration.

Parameters	Coefficient	95% CI	*P* value	*R*^2^
Gender	-0.629	-1.124, -0.134	**0.013**	0.204
Albumin (g/L)	0.058	0.023, 0.093	**0.001**	
Proton pump inhibitor	-0.828	-1.316, -0.340	**0.001**	

P values retaining significance in multiple linear regression analysis are shown.

The bold values indicate significant differences (p < 0.05).

### Factors Influencing Probability of the Therapeutic Window of PCZ for Prophylaxis

Based on the analysis results of the factors affecting the concentration variation of PCZ, we further analyzed the influence of these factors on the probability of the therapeutic window of patients receiving PCZ for prophylaxis. 142 measures (74.7%) had reached the target threshold of 0.76 µg/mL. Comparison of the characteristics between the two groups is summarized in **Table 5**. In line with previous analysis, C_min_ of patients with higher CRP level was more likely to be lower than 0.76 µg/mL (*P* = 0.049), and patients with relatively subtherapeutic concentrations had lower albumin level. In addition, the co-administration of PPIs could significantly decrease the probability of the therapeutic window compared with no co-administration of PPIs (*P* = 0.024).

**Table 5:** The factors influencing low trough PCZ concentration.

Parameters	PCZ concentration		*P* values
	Subtherapeutic	Therapeutic	
	<0.76 μg/mL (n = 48)	≥0.76 μg/mL (n = 142)	
Gender	32 (66.7%)	95 (66.9%)	0.555
Age (years)	33 (16–79)	33 (16–80)	0.434
BMI (kg/m^2^)	21.22 (16.42–28.73)	21.75 (16.05–27.72)	0.561
Nausea/emesis	10 (20.8%)	34 (23.9%)	0.264
Diarrhoea	2 (4.2%)	5 (3.5%)	0.579
Mucositis	2 (4.2%)	4 (2.8%)	0.487
CRP (mg/L)	13.04 (0.50–168.35)	4.85 (0.08–244.2)	**0.049**
Albumin (g/L)	34.8 (17.9–48.0)	38.8 (26.3–135)	**0.011**
GFR (mL/min)	105.69 (18.2–134.1)	117.0 (19.8–173.0)	0.572
ALT (U/L)	22 (4–853)	20 (1–310)	0.291
AST (U/L)	22 (6–640)	22 (8–144)	0.155
γ-GT (U/L)	72 (13–1553)	39 (5–600)	0.107
ALP (U/L)	81 (33–323)	80 (38–288)	0.528
TBIL (mmol/L)	13 (3.6–130.9)	16.8 (3.7–80.9)	0.701
Proton pump inhibitor	38 (79.2%)	75 (52.8%)	**0.024**
metoclopramide	7 (14.6%)	13 (9.2%)	0.235

Data is presented as n (%), or as median (range).

The bold values indicate significant differences (p < 0.05).

## Discussion

PCZ was initially approved as an oral suspension formulation in 2006, then delayed-release solid tablets and an intravenous formulation were approved in 2013 and 2014, respectively. Although these formulations have proved to be more effective in fungal prophylaxis without increasing side effects. PCZ oral suspension is still an important alternative to patients with swallowing difficulties in circumstances where the intravenous formulation is not available. However, the bioavailability of PCZ oral suspension is affected by various conditions, including erratic absorption, which is influenced by food, the concomitant use of medications, gut motilityand gastric absorption disorders. The present study provided real-world PCZ oral suspension concentration data and showed that significant variability in PCZ oral suspension C_min_ exists among different patients (**Figure 3**). The concentration may be affected by diarrhoea, body weight, male gender, and co-administration of PPIs (Miceli et al., 2015; Cojutti et al., 2018). All these factors make it difficult to attain optimal target concentrations. But factors that could potentially contribute to such variability remain to be fully elucidated. Additionally, Jeong et al. reported that escalating the PCZ suspension dose beyond 800 mg daily did not appear to result in consistent increases in C_min_ (Jeong et al., 2018). From this viewpoint, it seemed not be appropriate to predict the blood drug concentration completely based on the drug dose in order to achieve the therapeutic window.

In this study, 46 patients treated with PCZ suspension for prophylaxis of IFI were monitored. Logistic regression analysis revealed that the PCZ C_min_ value of 0.76 µg/mL was associated with >80% probability of successful therapy (Wang et al., 2014). Similar to a prospective study of PCZ prophylaxis of IFIs in patients with hematological diseases [the success rate was (64/74) 86.5%] (Li et al., 2020), and a randomized clinical trial for prophylaxis against IFIs [the success rate was (85/91) 93.5%] (Jang et al., 2010), the current study found that 83.3% of patients (30/36) successfully responded to PCZ prophylaxis. In agreement with the previous study (Chau et al., 2014), a PCZ C_min_ of 1.0 µg/mL was chosen as the lower limit to treatment of a proven, probable, or possible IFI, and the logistic regression model showed that this C_min_ was associated with a probability of successful response of 80.5% (**Figure 3B**). Our lower limit, however, is different from that previously published by Walsh et al. (2007). In that report, for patients with average plasma concentrations >1.25 µg/mL, clinical effectiveness was increased to 75%. In this analysis, a cut-off C_min_ was identified for patients as the concentration where successful clinical response increased >80% probability (Wang et al., 2014).

Similar to findings of Dolton et al. (2012) and Jeong et al. (2018), the present study found no relationship between PCZ C_min_ and hepatotoxicity, although the PCZ plasma concentrations were numerically higher among patients who undergone PCZ-related hepatotoxicity. The treatment for one patient who received PCZ suspension for prophylaxis fungal infection was seitched to carpofungin when alanine transaminase levels and aspartate transaminase levels increased more than five times the upper limit of normal. The PCZ concentrations in this patient ranged between 0.65–1.02 µg/mL. Cessation of PCZ led to gradual improvement of the liver functions. It is worth noting that Tverdek reported episodes of hyperbilirubinaemia when PCZ C_min_ is over 1.83 µg/mL, although not attributed to PCZ (Tverdek et al., 2017). In contrast, our study indicates that there is no a clear increase in hepatic damage when PCZ C_min_ is over 1.83 µg/mL. It is possible that concentration-toxicity relationship shows different profile among different races. Given that the relationship between PCZ C_min_ and adverse events has yet to be established, further surveillance is warranted. The relationship between PCZ concentrations and mortality rates could not be established since the cause of death could not be identified as fungal infections.

Additionally, we also investigated factors contributing to dose-normalized PCZ concentration variability. Dose-normalized PCZ concentrations were reported instead of actual concentrations to take into account the large variations in PCZ daily doses used when the samples were obtained (Allegra et al., 2017; Jeong et al., 2018; Märtson et al., 2019b). By using this measure, the estimated values could be compared within and between patients without taking variations in the dosage into consideration. No statistically significant differences were detected in age, BMI, digestive disorders, level of serum creatinine, level of γ-Glutamyl transpeptidase, level of alkaline phosphatase, as well as level of total bilirubin or co-administration of metoclopramide in our population. These factors have been analyzed in various studies, obtaining different results depending on the population analyzed (Desplanques et al., 2014; Jeong et al., 2018; Sime et al., 2019). Jeong et al., in agreement with our results, did not observe any association between plasma levels of PCZ and age, BMI, diarrhoea or nausea (Jeong et al., 2018). Conversely, Sime et al., analyzing adult critically ill patients from Australia, described statistically significant differences in concentrations depending on BMI (Sime et al., 2019). Dolton et al. reported coadministration with metoclopramide, underlying diarrhea or mucositis were predictors for lower C_min_ in haematological malignancies patients (Dolton et al., 2012). However, no such association was observed in our hematological disorders patient. Although PCZ treatment is associated with liver function abnormalities (Boglione-Kerrien et al., 2018), the effect of liver function on PCZ concentrations was not confirmed in our study, which is consistent with the results previously published by Märtson et al. (2019b). Collectively, these results indicate that it is difficult to predict PCZ concentration that we have to monitor trough concentration and adjust PCZ dosage according to the results.

Compared to patients administered PCZ alone, a 25.9% lower PCZ concentrations (*P* = 0.001) was noted in the patients receiving PCZ co-medicated with PPIs. In a cohort of 43 patients presenting with acute myeloid leukemia, Walsh and colleagues found co-administration with PPI significantly impact PCZ C_min_ (Walsh et al., 2007). A similar result was identified by Cojutti (Cojutti et al., 2018). After correcting the effects of other factors by multiple linear regression, co-administration of PPI was still a vital factor contributing positively to the variability of PCZ concentration. It is attributed to altered gastric pH caused by H_2_ receptor antagonist reducing PCZ absorption, which was confirmed through monitoring of intraluminal PCZ concentrations (Walravens et al., 2011).

Notably, out of the five most frequently recorded concomitant PPIs, lansoprazole and pantoprazole reduced dose normalized plasma PCZ C_min_ significantly in the univariate analysis. In contrast, omeprazole, the most frequently recorded proton pump inhibitor, showed only minor effects on PCZ concentration. Several controlled studies in healthy volunteers have documented 32% - 37% decrease in the area under the concentration-time curve (AUC) of PCZ with esomeprazole (Sansone-Parsons et al., 2007; Kraft et al., 2014). Nevertheless, an association between low PCZ concentrations and co-administration with esomeprazole (*P* = 0.179) was not observed in our study, which may be due to the paucity of concentration data of omeprazole combination.

Conflicting results exist regarding the effects of H_2_ receptor antagonist on PCZ. The product information states that no PCZ dosage adjustment is needed when co-administered with H_2_ antagonists other than cimetidine (Vehreschild et al., 2012). However, Dolton et al. found significantly lower PCZ concentrations in patients receiving H_2_ receptor antagonist ranitidine (*P* < 0.01) (Dolton et al., 2012). Unfortunately, due to the relatively small number of patients concomitant with H_2_ receptor antagonist, it is impossible for us to evaluate the impact of H_2_ receptor antagonist on PCZ C_min_ in the present study.

The univariate and multiple linear regression analysis both showed that lower PCZ concentrations were observed with decreasing albumin concentrations, which is in line with previous analysis (Märtson et al., 2019b; Sime et al., 2019). Although CRP level in the current study was negatively correlated with PCZ C_min_ in the univariate analysis, multiple linear regression analysis did not reveal any statistically significant association, which is consistent with the findings of Märtson et al. (2019a). Furthermore, in this study, CRP and albumin level were identified to be the risk factors for sub-therapeutic C_min_ (*P* = 0.049 and *P* = 0.011, respectively). Therefore, it is necessary to monitor PCZ therapy more frequently during inflammation.

Finally, another unexpected finding in our study is the association between PCZ concentrations and gender. Female patients were more likely to achieve higher PCZ levels in our study. This finding seems contradictory to some studies, of which greater PCZ concentrations in males than females were noted (Allegra et al., 2017; Jeong et al., 2018). On the contrary, no significant association was observed in other studies (Dolton et al., 2012; Märtson et al., 2019a; Oh et al., 2020). This could be due to gender-based differences in CYP-mediated metabolism, sexual hormone influence on drug absorption and differences in fat percent-age in body composition (Beierle et al., 1999). If confirmed in later studies, these findings may help to explain inconsistent results, but more researches incorporating the potential mechanisms should be on the way.

There are several limitations in our study. A limitation of this study is the only limited number of cases, especially in the sub-population analyses, making the results less representative. Confounding factors known to influence PCZ concentrations, e.g., food effects, co-administration of phenytoin and rifampicin or genetic polymorphism of *P-gp* or *UGT* (Jeong et al., 2017; Suh et al., 2018), were not further explored in our study attributed to the relatively small sample size. More studies are warranted to further clarify the role of these factors in PCZ variability.

## Conclusions

The present study determines the high inter- or intra-patient variability in PCZ C_min_ in patients with hematological disorders who receive PCZ oral suspension. Gender, albumin concentration and co-administration of PPI were independent factors affecting PCZ Cmin. CRP and co-administration of PPI significantly decreased C_min_ and probability of the therapeutic window providing treatment benefits. It is necessary to adjust the dosing regimens based on PCZ C_min_ to obtain an optimal therapeutic response.

## Data Availability Statement

The original contributions presented in the study are included in the article/supplementary material; further inquiries can be directed to the corresponding authors.

## Ethics Statement

The study was reviewed by the Human Research Ethics Committee of The First Affiliated Hospital of Zhengzhou University and Ethics approval was obtained (No. 2020-KY-171). Informed consent was waived due to the retrospective nature of this study.

## Author Contributions

X-JZ and JY designed the clinical trial. M-MJ, Q-WZ, and Z-FQ performed the drug concentration analyses. R-QL and X-KT performed the clinical data collection. M-MJ performed the statistical analyses. All authors contributed to the article and approved the submitted version.

## Funding

This work was supported by National Natural Science Foundation of China (81803638, 81903704) and Henan Provincial Department of Science and Technology Research Project (182102310330).

## Conflict of Interest

The authors declare that the research was conducted in the absence of any commercial or financial relationships that could be construed as a potential conflict of interest.
